# Targeted lipid nanoparticles to prevent trans-placental passage in the ex vivo human placental cotyledon perfusion model

**DOI:** 10.1007/s13346-024-01715-6

**Published:** 2024-10-14

**Authors:** Caren van Kammen, Hedwig van Hove, Dimitrios Kapsokalyvas, Rick Greupink, Raymond Schiffelers, Titia Lely, Fieke Terstappen

**Affiliations:** 1 Department of Nanomedicine, LAB CDL Research, UMC Utrecht, Utrecht, The Netherlands; 2 Department of Pharmacy, Division of Pharmacology and Toxicology, Radboud UMC, Nijmegen, The Netherlands; 3 Department of Genetics and Cell Biology, Maastricht University, Maastricht, The Netherlands; 4 Interdisciplinary Centre for Clinical Research IZKF, University Hospital RWTH Aachen, Aachen, Germany; 5 Department of Obstetrics, Wilhemina Children’s Hospital, UMC Utrecht, Utrecht, The Netherlands; 6 Department of Neonatology, Wilhemina Children’s Hospital, UMC Utrecht, Utrecht, The Netherlands

**Keywords:** Nanomedicine, Drug delivery system, Pregnancy, Placenta transport prevention, Placenta (perfusion)

## Abstract

Medication use during pregnancy poses risks to both the mother and the fetus. These risks include an elevated potential for fetotoxicity due to placental drug transport. Nanomedicines offer a promising solution by potentially preventing trans-placental passage. Targeted nanomedicines could enhance safety and efficacy in treating maternal or placental pathophysiology. Our study investigates placental transfer kinetics of targeted lipid nanoparticles (LNPs) in an ex vivo human placenta cotyledon perfusion model. We collected human placentas for dual-side ex vivo placental perfusions. Targeted LNPs with a fluorescence tag were introduced into the maternal circuit of each placenta. To establish if there was trans-placental passage of LNPs to the fetal circuit, we collected samples from maternal and fetal circuits throughout the six hours of the perfusion. We determined the fluorescence signal using a multi-mode microplate reader and Multiphoton microscopy to localize the LNPs in the placenta tissue. Data from perfused placenta tissue showed no significant transfer of the fluorescently labeled LNPs across the placental barrier to the fetal circuit. Localization of targeted LNPs in tissue samples is mainly observed in the maternal blood space of the placenta. Our results suggest that targeted LNPs present a promising strategic approach to prevent trans-placental passage to the fetus. Our future perspectives involve investigating the efficacy of targeted LNPs as well as loading targeted LNPs with nucleic acid-based therapeutics to investigate their therapeutic potential.

**Graphical Abstract:**

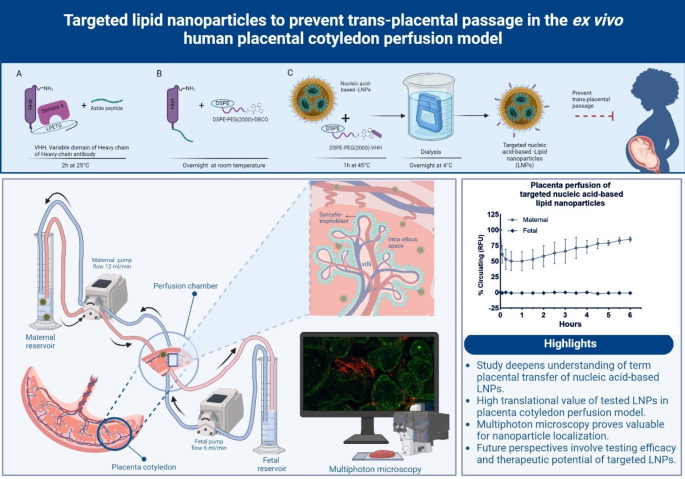

**Supplementary Information:**

The online version contains supplementary material available at 10.1007/s13346-024-01715-6.

## Highlights

Study deepens understanding of term placental transfer of nucleic acid-based LNPs.

High translational value of tested LNPs in placenta cotyledon perfusion model.

Multiphoton microscopy proves valuable *to localize* nanoparticles.

Future perspectives involve testing efficacy and therapeutic potential.

**Supplementary Information:**

The online version contains supplementary material available at 10.1007/s13346-024-01715-6.

## Introduction

Therapy of a (chronic)disease during pregnancy such as mental disease, diabetes, thrombosis (prophylaxis), epilepsy, and chronic kidney diseases often require medication. This includes diseases that arise during pregnancy, such as hypertensive disorders involving placental pathophysiology (including preeclampsia). Approximately 90% of the medications present challenges, as they are mostly used off-label and have not been specifically tested for safety during pregnancy. Medication use during pregnancy may adversely affect the mother and fetus, including the risk of fetotoxicity. This is because most medicines are small molecules (< 600 Da) that passively transport over the placental barrier to the fetus [1].

Nanoparticles could prevent such passive or facilitated diffusion. Nanoparticles are colloidal systems and defined as particles below one micrometer. When the nanoparticle encapsulates drug molecules, the fate of the drugs no longer depends on its own characteristics. Tailoring the physicochemical characteristics of nanomedicine, such as the composition, size, and surface charge, alters the tissue distribution profile and can steer where encapsulated therapeutics are released [2]. Decorating the surface of the nanomedicine with targeting ligands enhances the efficacy of the drug by specifically interacting with desired cell types [3–6]. In the context of hypertensive disorders of pregnancy, targeting is particularly aimed at endothelial or trophoblast cells. Nanomedicine offer exciting potential as a therapeutic strategy during pregnancy by controlling the drug interaction with the placenta and enhancing safety by preventing exposure of drugs to the fetus.

*Lipid nanoparticles* (LNPs) are currently the leading non-viral delivery systems for nucleic acid-based therapeutics, including siRNA, mRNA, or plasmid DNA [7]. These LNPs are utilized for disease treatments by either silencing pathogenic genes or expressing therapeutic proteins. To overcome challenges of nucleic acid-based therapeutics, for instance rapid clearance from circulation and susceptibility to degradation, various strategies such as chemical modifications or encapsulation in nanoparticles are employed. Nucleic acid-based therapeutics hold promise in the obstetric field, as indicated by a study in baboons that showed a positive impact of siRNA delivery by a targeting peptide on the phenotype of preeclampsia [8]. Furthermore, nucleic acid-based therapeutics encapsulated in LNPs decorated with VHH nanobody (Variable domain of Heavy chain of Heavy-chain antibody) enhance the specificity of target tissue [9, 10]. The use of LNPs during pregnancy remains uncharted territory in which especially the optimal and safe design of LNPs to specifically prevent trans-placental passage needs further elucidation [2].

This study aims to determine whether targeted LNPs with VHH nanobody, against specific epitopes found in endothelial and placental tissue, prevent trans-placental passage in an ex vivo human placenta perfusion model. The majority of studies evaluate nucleic acid delivery during pregnancy in an animal model, especially mice [6, 11–13]. Although in vivo animal models allow for the evaluation of efficacy, immunogenicity, and toxicity, mice placenta have physiological and anatomical differences compared to human placentas [14]. Therefore the ex vivo human placenta perfusion model offers a high translational value and detailed investigations of the placental passage of medicine or drug delivery systems in the human placenta without considering the ethical concerns [15]. We use multiphoton microscopy (MPM) as a sophisticated imaging technique to localize the fluorescently labeled targeted LNPs in the placenta tissue without extra staining of the tissue, neither cutting it into thin slices. The outcome of this study would enable us to load nucleic acid-based therapeutics into our targeted LNPs, aiming to enhance maternal and placental health in obstetric diseases such as preeclampsia and fetal growth restriction. Moreover, this design could be rapidly adapted for other therapeutic approaches during pregnancy by decorating various VHH nanobodies against different target tissues and thereby applied across a broad spectrum of pregnancy complications.

## Methods

### Targeted lipid nanoparticle development

We prepared LNPs by microfluidic mixing using the NanoAssemblr Benchtop (Precision Nanosystems, Vancouver, Canada). An ethanolic phase containing lipids was mixed with an acidic aqueous phase (25 mM sodium acetate, pH 4.0) containing nucleic acids leading to the formation of LNPs. Lipids were dissolved in 100% ethanol at a total lipid concentration ranging from 20 mM. LNPs were composed of DLin-MC3-DMA VKB (India), cholesterol Sigma Aldrich (Saint Louis, MO, USA), DSPC Lipoid, (Ludwigshafen am Rhein, Germany), and PEG-DMG lipid NOF Corporation (Tokyo, JP),.at a molar percentage of 50, respectively.

The nucleic acids mixture underwent encapsulation at a nitrogen-to-phosphate ratio (N/P) of six. DSPE-PEG- Cy 5.5 (0,2% Mol of total lipid concentration) was used for fluorescent labeling of LNPs. LNPs emerged through production at a total flow rate (TFR) of 4 ml/min and a flow rate ratio (aqueous phase; organic phase) of 3 to 1. Immediately after production, LNPs underwent dialysis against an excess of phosphate-buffered saline using Slide-a-Lyzer™ dialysis cassettes G2 with a membrane cutoff of 10.000 Da (Thermo Scientific, Waltham, MA, USA).

### Click chemistry conjugation of VHH nanobody

Our lab developed nanobodies for five different markers (VCAM-1, fibrin, aVWF, Thrombomodulin, TFPIβ) against vascular endothelial targets, and one VHH as a placental trophoblast target (folate alpha) was designed. These VHH nanobodies can potentially serve as diagnostic targets for various vascular diseases. The development of VHHs was performed as previously described [16]. Further details on VHH selection and click chemistry can be found in the supplemental method section.

### Characterization of the targeted LNP

Dynamic light scattering (DLS) analyses were performed to determine the hydrodynamic diameter of LNPs using a Zetasizer Nano S (Malvern Panalytical, Malvern, UK) equipped with a 4 mW HeNe laser of 633 nm. We prepared LNP samples using PBS pH = 7.4 and we measured the scattering at an angle of 173º at 37 ºC for 10 s and repeated at least 10 times. Z-average sizes of three sequential measurements were collected and analyzed on size and polydispersity (PDI). PDI represents the distribution of size populations within a sample, ranging from 0.0 (perfectly uniform) to 1.0 (highly polydisperse). In lipid-based drug delivery, like liposomes, a PDI of 0.3 or lower indicates a homogeneous population of phospholipid vesicles [17]. Zeta potential was measured by laser Doppler electrophoresis on a Zetasizer Nano Z (Malvern Panalytical, Malvern, UK). Samples were diluted in 0.1 × DPBS and measurements of three replications were carried out at 25 °C.

The encapsulation efficiency of LNPs measured with the Ribogreen assay calculated using the following formula: Encapsulation Efficiency (%) = amount of DNA in LNP / the total amount of Barcode DNA × 100%.

The stability of LNPs of the encapsulated Barcode DNA from the LNPs was evaluated using the dialysis method in nuclease free PBS (pH 7.4). The LNPs were placed into dialysis cassete, which were then immersed in medium within a shaking incubator set at 37 °C and an oscillation speed of 100 rpm. At designated time points (0, 0.5, 1, 4, 6, 24, and 30 h), samples were taken from the dialysis buffer, and the amount of free Barcode DNA was analyzed with ribogreen assay. The stability of percentage encapsulated Barcode DNA was calculated as the Encapsulation Efficiency minus the percentage of released barcode DNA.

To assess the particle size of the LNPs in an in vitro environment, a Krebs-Henseleit buffer supplemented with human albumin, was prepared as the dispersion medium for diluting the LNPs. The size stability study was conducted at 37 °C, with 0.5 mL of the LNP suspension sampled at predetermined time points (0, 0.5, 1, 2, 4 and 6 h). The samples were dialyzed overnight using 100 kDa cassettes to eliminate interfering peaks from the media. The particle size measurements were performed as previously described, and the experiment was conducted in triplicate.

### Placenta collection and informed consent

Placentas from uncomplicated singleton pregnancies were obtained from consenting women who underwent an elective cesarean section or vaginal delivery at the Radboud University Medical Center, Nijmegen, the Netherlands (approval of our institutional medical ethical committee was obtained, CMO Arnhem/Nijmegen File 2014 − 1397 and 2022–13523). Clinical details are presented in Table 1. After birth, the placentas were kept at room temperature and were transported to the laboratory immediately and weighted. Placenta efficiency ratio (PER) was calculated with the following formula PER = fetal weight (g)/ placental weight (g). Placental efficiency refers to the functional capacity of the placenta in supporting fetal development by facilitating the transfer of essential nutrients and oxygen from the maternal to the fetal circulation. In a healthy pregnancy, the placental efficiency ratio typically ranges from 6:1 to 9:1 [18, 19].

**Table 1:** Clinical characteristics of patients of used placentas

Maternal characteristics					Labor characteristics		Child characteristics			Placental characteristics	
Age (years)	BMI (kg/m^2^)	Primi- parous	BP (mmHg)	Medication	GA at delivery (weeks + days)	Mode of delivery	Birth weight (grams)	Head circumference (cm)	Gender	Placenta weight (grams)	PER
34	21	No	129/80	etoricoxib codeine	38 + 5	CS	3390	*35.9*	Male	544	*1:6*
35	24	Yes	115/82	certolizumab pegol	39 + 5	Vaginal	3156	*36.0*	Female	350	*1:9*
41	28	No	123/78	N/A	38 + 6	CS	3490	*35.5*	Female	602	*1:6*

footnote None of the women smoked. CS, cesarean section; BP, blood pressure; GA, gestational age; N/A, not applicable; PER, Placetnal efficiency ratio

### Ex vivo human placenta cotyledon perfusion experiment

The perfusion procedure followed the method outlined by Schneider et al. [20, 21], with minor modifications reported by Bukkems et al. [22]. In brief, placenta perfusion of an intact cotyledon was started within 45 min after delivery by canulation of the matching fetal vein and artery (6 mL/min) of a single cotyledon.

To represent maternal circulation, four cannulas were inserted through the maternal decidual plate into the intervillous space (12 mL/min).

Initially, both circulations were left open to wash out residual blood for a minimum of 45 min. The washing buffer consisted of Krebs-Henseleit buffer, supplemented with 11.1 mM glucose and 2500 IU/L heparin (LEO Pharma, Amsterdam, The Netherlands). After wash-out, the solutions were changed to experimental buffers consisting of the same buffer supplemented with human albumin (maternal 34 g/L and fetal 29 g/L) (Albuman^®^, Sanquin, Nijmegen, the Netherlands), and circulations were closed. The maternal buffer was gassed with 95% O_2_/5% CO_2_ and the fetal buffer with 95% N2/5% CO2 while being kept at 37 °C at a pH between 7.2 and 7.5. Before infusion baseline samples were collected.

The fluorescent-labeled LNPs (100ug/ml, 1 mL, mixture of all VHH targets) and antipyrine (100 mg/L) were added to the closed maternal circulation, which had a total volume of 120 mL. Samples were taken at fixed time points from the maternal and fetal circulation and were stored at − 20 °C until analysis. At the end of the experiment, the placentas were flushed for thirty minutes with PBS after the perfusion of the LNPs. The perfused cotyledon was then cut into pieces and frozen in methyl butane on dry ice and stored at − 80 °C. Frozen placenta tissue was sectioned into 200 μm slices to perform multi-photon microscopy and into 10 μm for hematoxilin and eosine staining as an objective observation of placenta health after pefusion.

### LNP determination in maternal and fetal circulation

The ability to prevent the transplacental transfer of the LNP was indirectly assessed by measuring the concentration of Cy5.5 in the sample. Cy5.5 fluorescence intensity of maternal and fetal circulation samples was measured (excitation/emission 680/710 nm) using a microplate reader (Spectramax ID5). Measurement of Cy5.5 concentration relied on a pre-established calibration curve, normalized to % of timepoint 0, and expressed as % circulating LNP in RFU.

### Antipyrine determination by LC-MS/MS

For every placental perfusion, the control substance Antipyrine (molecular weight: 188 Da, logP: 0.38) was added to the maternal circulation (100 mg/L). It undergoes rapid passive diffusion across the placental barrier and was used to confirm maternal and fetal circulation overlap.

The collected samples were analyzed at the laboratory of the Department of Pharmacy, division of Pharmacology and Toxicology, Radboud University Medical Center. Antipyrine measurements were performed using an LC-MS/MS method with an Acquity UPLC (Waters, Milford, MA, USA) coupled to a Xevo TQ-S micro (Waters) triple quadrupole mass spectrometer. The internal standard d3-antipyrine (Toronto Research Chemicals) was used. Protein precipitation was achieved by the addition of acetonitrile to the samples in a ratio of 3:1. After vortexing, samples were centrifuged at 13,000 rpm for 3 min. Samples were injected on an Acquity UPLC HSST3 column and separation was accomplished via gradient elution (flowrate 0.300 mL/min). Further details about LC/MS can be found in supplemental methods.

### Localization of LNPs using multi-photon microscopy

Images were acquired with the multiphoton microscope Leica STELLARIS 8 DIVE FALCON (Leica Microsystems GmbH, Wetzlar, Germany). Samples were mounted on slides with a drop of PBS and cover slip. Fluorescence was collected with a 25x Leica HC IRAPO, NA 1.0 water motCORR collar objective. Autofluorescence was excited at 780 nm and detected between 450 and 520 nm. Cy5,5 signal from LNP’s. was excited at 1300 nm and detected between 670 and 730 nm. The images were obtained using 8-bit in bidirectional scanning mode. The typical field of view was 591 × 591 µm^2^ with 1024 × 1024 pixels and the imaging depth ranged around 150 μm, with variations of ± 50 μm between stacks and samples. The Z-step size varied from 1.5 μm to 5 μm. The voxel size was typically 0.57 × 0.57 × 3 µm^3^.

### Statistical analysis

All data are presented as mean ± standard deviation (SD). Each perfusion sample from every placenta was measured in duplicate. Data from three placentas was utilized in GraphPad Prism software to perform a two-way analysis of variance (ANOVA) followed by Tukey’s Multiple Comparison Test. Means were considered statistically significant in case of a two-sided p-value < 0.05. P-values were categorized as *p < 0.05, **p < 0.01, ***p < 0.001 and ****p < 0.0005.

## Results

### Profiling of targeted LNPs

After production of the LNPs the particle size measured on average 110 nm with a polydispersity index value of 0.14. This indicates that the LNPs have a tight size distribution. The encapsulation efficiency was determined to be 91.1% ± 1.4% on average. The surface charge was measured from a separate batch, yielding a mean value of -3.0 mV, which is near neutral. Stability of encapsulation and size of LNPs from a separate batch are presented in Figure S3.

### Prevention of transplacental passage of targeted LNPs

**Fig. 1:**
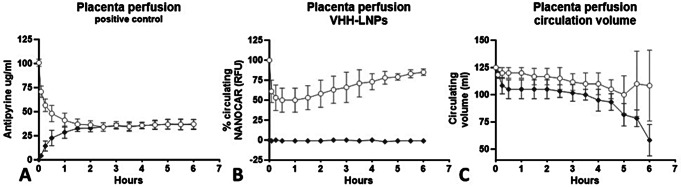
(**A**) Concentration–time profile of antipyrine (100 mg/L) in the maternal (open circles) and fetal circulation (closed diamond shapes), based on perfusion of three placentas for 360 min. (**B**) Concentration of LNPs in the maternal circulation (open circles) and fetal circulation (open diamond shapes) after perfusion with selected nanobodies labeled with Cy5.5 fluorophore. All data points *p* < 0.0005. (**C**) Volume distribution over time. *A decline in maternal and fetal circulation volumes in the final two hours of perfusion likely corresponds to observed edema in the placental cotyledon.* Data are depicted as mean ± SD. LNP, Lipid nanoparticle; VHH, Variable domain of Heavy chain of Heavy-chain antibody

Clinical characteristics of the three used placentas in the placenta perfusion experiments are shown in Table 1. All monitored biochemical and physiological data were within specifications for human placental perfusions (Table S1). The LNP and antipyrine were added to the maternal perfusate after the wash period and the kinetics of placental transfer from maternal to fetal side was evaluated. The marker antipyrine crossed the placenta rapidly, and equilibrium was reached after 90 min (Fig. 1a). The antipyrine values in fetal circulation remained steady until the end of perfusions. The LNP concentration in the maternal circuit decreased to an average of 50% of the infused dose in the first 30 min (Fig. 1b). This was followed by an increase to a maximum of the average of 86% of the infused dose.

The nanomedicine did not appear in the fetal circulation over the whole period of six hours of perfusion. We observed a decline in volumes of maternal and fetal circulation in the last two hours of perfusion (Fig. 1c). Possibly this is in relation to the edema in the placental cotyledon which was observed by visual inspection. For an objective evaluation of placental health after perfusion, hematoxylin and eosin stained slides are presented in Figure S1.

### Localization of the targeted LNPs

To cross the transplacental barrier from maternal to fetal circulation the LNPs would have to traverse two barrier cell layers, the villous syncytiotrophoblast (SCT) and the endothelial cells (EC) that line the fetal capillaries inside the core of villous tissue. The core of villous tissue comprises various components including fetal capillaries and connective tissue fibers like collagen. The maternal inter-villous space surrounds the villous tissues.

**Fig. 2:**
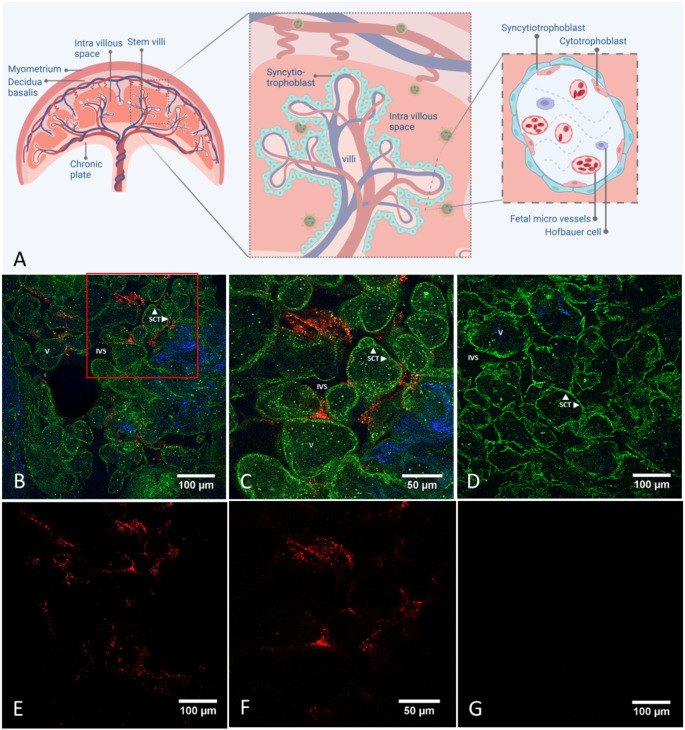
Placental villi imaged with MPM. **A**) Schematic overview of placental tissue with focus on the localization of LNPs within the maternal intravillous space. **B-C**) Representative images of a perfused placental villi with fluorescent signal (Cy5.5) of labeled LNPs (red) visible in maternal inter-villous space (black), placental villi (green), and fibrillar collagen (blue) in the core of placental villi. **C**) Magnified red lined area of image a **D**) Control placental villous tissue of nonperfused tissue E-F-G) The red channel of B-C-D correspondingly. LNPs, Lipid nanoparticles; MPM, multiphoton microscopy; SCT, villous syncytiotrophoblast; V, core of villous tissue

Multi-photon fluorescence microscopy was used to visualize the distribution of LNPs within the villous tissues from each perfused placenta. The presence of nanoparticles (coded red) in the maternal inter-villous space can be observed around the border of the syncytiotrophoblast in the inter-villous space (Fig. 2). Occasionally the lipid nanoparticle appears to be internalized in the outer border of the syncytiotrophoblast cells.

## Discussion

The fluorescently labeled LNPs were not observed in measurable quantities in the fetal perfusate of the ex vivo perfused healthy term placentas. The MPM imaging revealed no localization in fetal villous capillaries which confirms the results of the sample analyses of placental perfusion with no transport of LNP to the fetal circuit.

### Transplacental passage of LNP in the placenta cotyledon perfusion model

To our knowledge, we are the first to demonstrate that targeted LNPs, used as a delivery system for nucleic acid therapeutics during pregnancy, prevent transplacental passage in the perfused human placenta model.

The observed variance in the sample analyses of the maternal distribution could be attributed to inter-placental differences and the size differences of the selected cotyledon. The observed decrease in the number of LNPs in the first 30 min can be partially attributed to the dilution of the maternal reservoir with the volume of remaining washing buffer inside the maternal circuit. Furthermore, this decrease could be due to the targeting of the LNP temporarily to placental tissue.

The visible decrease in volume after three hours and the placenta containing more fluid could be a result of cell swelling. Cell swelling is a complex process influenced by several factors [23]. Prolonged perfusion can lead to an imbalance in the movement of fluids and solutes across cell membranes, resulting in osmotic stress and subsequent cell swelling. Similarly, inflammatory conditions can cause the release of cytokines and other inflammatory mediators, disrupting normal cellular function and leading to changes in osmolality that contribute to cell swelling. As LNPs are widely used due to their established safety profile, as evidenced by their role in COVID-19 vaccines [24, 25]. However, the safety observed with LNPs in COVID-19 vaccines may not be generalizable to other administration routes or non-vaccine applications. Recent research has shown that LNPs activate the innate immune system across various administration routes [26–28]. This activation leads to the secretion of pro-inflammatory cytokines and chemokines, which facilitate the infiltration of activated leukocytes. Thereby causing tissue damage and lead to the cell swelling. Whether the cell swelling is a delayed effect of LNPs, only becoming visible at the end of the perfusion period, or a result of the relatively long perfusion period (6 h) itself, remains uncertain. Earlier studies investigating the disposition of biologics, also lasting for 6 h, did not report notable placenta edema taking place [29].

### MPM: a unique imaging technique to localize LNPs in complex placental tissue

The visualization of LNPs is challenged by the complex structure of the placenta. While other studies apply immunofluorescence techniques to localize particles in the placenta [30–32], we used the autofluorescence signal of the tissue excited by MPM as a sophisticated imaging technique. The main advantage of this technique compared to other optical imaging methods lies in its ability to penetrate deeper into the sample, eliminating the need to slice the tissue thinly. Therefore the 3D morphology of the tissue is preserved. Imaging of the villi is based on their inherent autofluorescence signal, therefore there is no need for complex staining steps to the standard immunofluorescence technique [33]. This is particularly valuable in localizing the particles throughout a larger area in depth of tissue. Furthermore, processing of tissue for immunofluorescence staining can interfere with the existing fluorescent label of nanoparticles. Our study without extra stainings indeed showed a clear image of the borders of the syncytiotrophoblast villous tissue and the presence of LNPs with Cy5.5 fluorescent label in the maternal inter-villous space.

### Strengths and limitations

Given that there is limited research focused on LNPs in human pregnancy, the present study enhanced our understanding and translational value of the transfer of nucleic acid-based LNPs across the placental barrier from mother to fetus. Specifically, as a therapeutic approach to target maternal or placental aspects with VHH nanobodies to guide the loaded LNPs to diseased tissues. The perfused human placenta model mimics the in vivo environment of the human placenta in (late) third trimester most efficiently. Therefore, this ex vivo model holds a high translational value and is widely regarded as the gold standard for testing drugs [15].

We acknowledge some limitations. Firstly, the success rate of placenta cotyledon perfusion for over 6 h was around 25%, therefore this study includes a small study group. Secondly, we cannot disprove the direct relation between LNPs and cell swelling in this data set. In the current study no control perfusions were conducted in which LNPs were not administered. Thirdly, we note that our data cannot be generalized to placentas in the first and second trimesters, as it is known that placental transfer varies with gestational age. Early pregnancy typically has higher placental permeability than at term and must be studied separately [34, 35]. While both are beyond the scope of this study, placental transfer could also be affected by pathological diseases where the capillary permeability increases under inflammatory conditions [36, 37]. Therefore, this ex vivo human placenta perfusion model of healthy placentas inevitably unqualifies for determining the most optimal VHHs targeting for dysfunctional placental syndromes considering the different placental expression between healthy and dysfunctional placenta.

## Conclusion and future perspectives

We conclude from our results that the targeted LNPs as a nucleic acid drug nanocarrier prevent trans-placental passage after a six-hour perfusion in the ex vivo human healthy term placenta. The perfusion and localization data suggest that our targeted LNPs could be an effective therapeutic strategy during pregnancy to prevent crossing the placental barrier from mother to fetus. We demonstrated that MPM imaging technique forms a useful tool for localization of fluorescently labeled nanoparticles in research. Future research needs to be performed on transplacental passage during earlier stages of gestation to confirm our findings preventing crossing the placental barrier in late gestation. Secondly, assessing the efficacy of the targeted LNPs in dysfunctional placentas with altered vascular conditions. Under these conditions of increased capillary permeability, targeting specific cell types in diseased tissue by the VVHs could play a more significant role in preventing transplacental passage and enhancing therapeutic efficacy. Furthermore, subsequent research should explore the therapeutic potential of the loaded therapeutic nucleic acids into LNPs, particularly in disease models such as preeclampsia and fetal growth restriction. Lastly, these LNPs can be customized and assessed for their effectiveness in managing other pregnancy complications. Overall, targeted LNPs may provide a promising approach for site-specific delivery of nucleic acid-based therapeutics to the mother without significant transfer to the fetus.

## Electronic supplementary material

Below is the link to the electronic supplementary material.

Supplementary Material 1

## Data Availability

The datasets generated during and/or analysed during the current study are available from the corresponding author on reasonable request.
