# Supplementary material for: Targeted lipid nanoparticles to prevent trans-placental passage in the ex vivo human placental cotyledon perfusion model
Source: Drug Deliv Transl Res. 2024 Oct 14;15(6):1985–93. doi: 10.1007/s13346-024-01715-6 (PMC12037421; doi:10.1007/s13346-024-01715-6)
Supplement: Supplementary file 1 — Supplementary Material 1 [file 13346_2024_1715_MOESM1_ESM.docx]

**Targeted lipid nanoparticles to prevent trans-placental passage in the *ex vivo* human placental cotyledon perfusion model**

Caren van Kammen^1^, Hedwig van Hove^2^, Dimitrios Kapsokalyvas^3,4^, Rick Greupink^2^, Raymond Schiffelers^1^, Titia Lely*^5^, Fieke Terstappen*^5,6^

*Contributed equally to this work

^1^ Department of Nanomedicine, LAB CDL Research, UMC Utrecht, Utrecht, The Netherlands.

^2^ Department of Pharmacy, Division of Pharmacology and Toxicology, Radboud UMC, Nijmegen, The Netherlands.

^3^ Department of Genetics and Cell Biology, Maastricht University, Maastricht, The Netherlands.

^4^ Interdisciplinary Centre for Clinical Research IZKF, University Hospital RWTH Aachen, Aachen, Germany.

^5^ Department of Obstetrics, Wilhemina Children’s Hospital, UMC Utrecht, Utrecht, The Netherlands.

^6^ Department of Neonatology, Wilhemina Children’s Hospital, UMC Utrecht, Utrecht, The Netherlands.

Supplemental material

**Table S1. Physiological data were within specifications for human placental cotyledon perfusions**

| **Specifications for human placental cotyledon perfusions** | | | | | |
| --- | --- | --- | --- | --- | --- |
| **Placenta #** | **pH maternal** | **pH fetal** | **Maternal leak observation** | **Outflow measured through fetal circuit at start of perfusion** | **Outflow measured through fetal circuit at start of perfusion** |
| 1 | 7.31±0.05 | 7.32±0.07 | No fluid leak visible | 6 ml/min | 6 ml/min |
| 2 | 7.37±0.07 | 7.38±0.07 | No fluid leak visible | 6 ml/min | 5.9 ml/min |
| 3 | 7.39±0.09 | 7.44±0.09 | No fluid leak visible | 6 ml/min | 6 ml/min |

Data of maternal and fetal pH is depicted as mean ± SD.


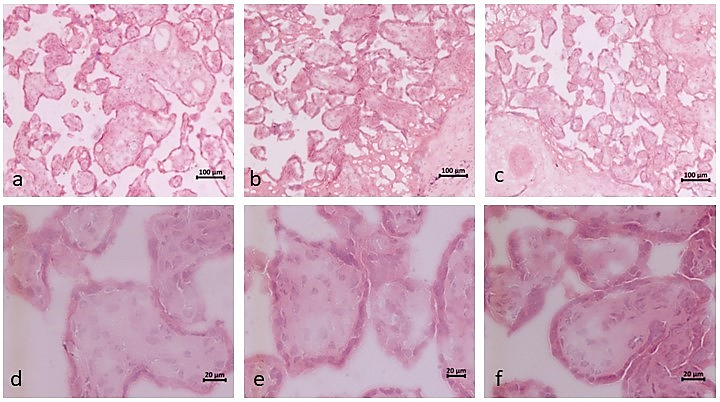
**Figure S1. a-b-c)** Objective observation of Hematoxylin and Eosin stained perfused placenta’s, 10x objective **d-e-f** The 40x objective of a-b-c correspondingly.


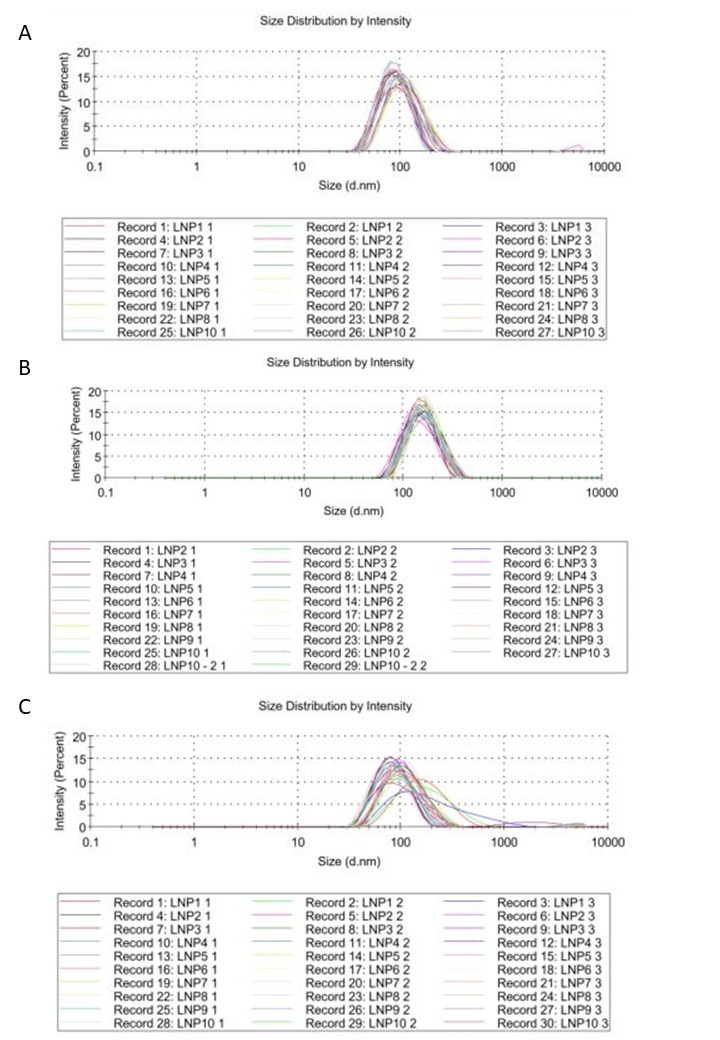


**Figure S2.** DLS histograms **A)** Size distribution Batch 1 injected in placenta 1 **B)** Size distribution Batch 2 injected in placenta 2 **C)** Size distribution Batch 3 injected in placenta 3.

**
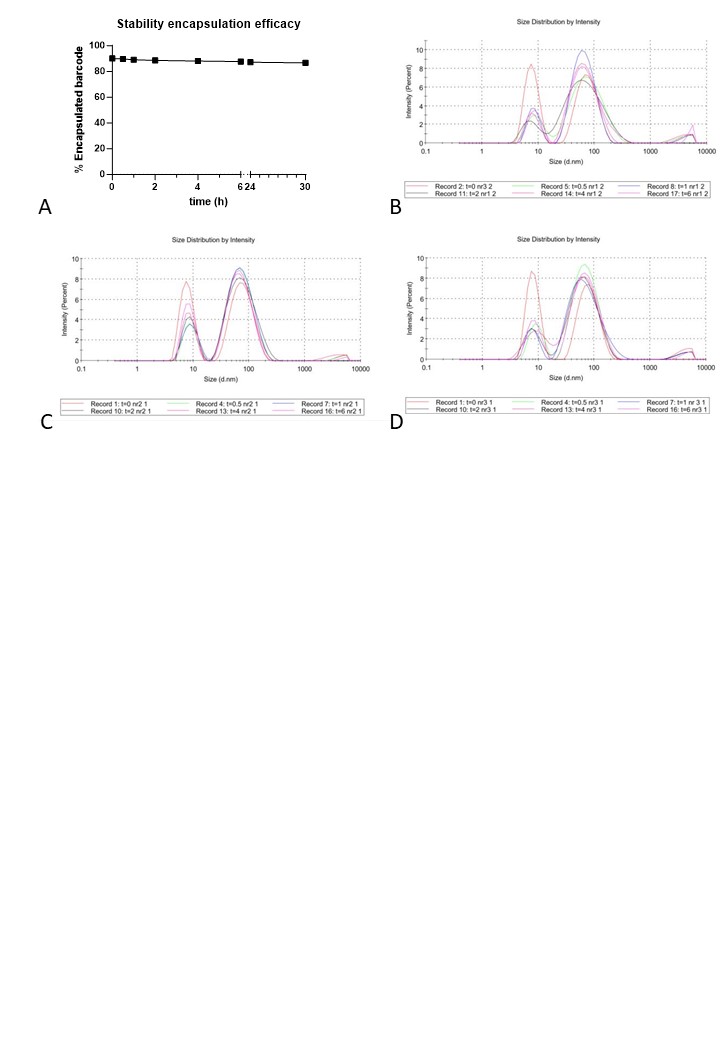
**

**Figure S3.** **A)** Evaluated stability of LNPs of the encapsulated Barcode DNA by dialysis method in nuclease free PBS (pH 7.4). **B-C-D)** Assessment of particle size stability of the LNPs in an in vitro environment conducted in triplicate. The particle size of the LNPs was measured at approximately 92 nm, with minor rest interference observed from media peaks at around 9 nm and >1000 nm after dialyzes.

**Movie S1**. 3D movie of MPM

Kammen, Caren (2024). 3D Movie Multi-photon microscopy of localization of the targeted LNPs. figshare. Media. <https://doi.org/10.6084/m9.figshare.26980849>

Supplemental methods

Click chemistry conjugation of VHH nanobody

In short, two Llama glama were immunized with specific protein degradation products over four rounds at weekly intervals. Following this, a bacteriophage library was created which contained only the heavy chain-antibody repertoire, phage display was used to obtain specific VHHs. The VHH insert was ligated into the pTH4.Click production vector. VHHs were purified with immobilized metal affinity chromatography (IMAC) on Talon Superflow Sepharose (Cytiva, Marlborough, USA). Lastly, the purified VHHs were passed through a Superdex 200 pg column for size exclusion chromatography (Cytiva, Marlborough, USA) and preparations were >95% pure as confirmed by the use of SDS-Page and Coomassie blue staining.

Enzymatic addition of Gly3-Lys-azide took place at the C-terminus of the VHHs. Performed as follows, substances were incubated (50 μM VHH containing an LPETG tag, 250 μM Gly3-Lys-azide, and 2,5 μM Sortase A in tris-buffered saline (TBS; 50 mM Tris, 150 mM NaCl, pH7.4) for 30min at RT. The resulting VHH-azide intermediates were subjected to IMAC on Talon Superflow to remove Sortase A and unmodified VHHs, followed by size exclusion chromatography on Zeba spin 7kDa desalting columns to remove excess Gly3-Lys-azide. Subsequently, the VHH-azide intermediates reacted with DBCO-conjugated acceptor molecules, including DBCO-PEG (20kDa). Reactions were performed at a VHH concentration of 80 µM and we removed Excess DBCO with Zeba spin 7kDa desalting columns. VHH nanobodies were clicked to the LNPs with post-insertion method. The components (VHH+DBCO-PEG-DSPE +lipid mix) incubated at 42 degrees Celsius for 2 hours. Followed by, a dialyze overnight to remove unbound VHH to PBS with micro float dialyze (float alyzer) 100 kD.

Antipyrine determination by LC-MS/MS

Gradient conditions were as follows: 0-1.0 min, 100% solvent A and 0% solvent B; 1-3 min, 100% solvent B and 0% solvent A. Solvent A consisted of 100% water and 0.1% formic acid, solvent B of 100% acetonitrile and 0.1% formic acid. The effluent from the UPLC was passed directly into the electrospray ion source. Positive electrospray ionization was achieved using nitrogen as a desolvation gas with an ionization voltage of 0.3 kV. Furthermore, argon was used as a collision gas and the source temperature was set at 500 °C.

Detection of the compounds was based on the isolation of the protonated molecular ion and subsequent MS/MS fragmentations and multi-reaction monitoring (MRM) was carried out. The following MRM transitions were used: for antipyrine, m/z 188.98 (parent ion) to m/z 55.83 and 76.96 (both product ions), and for d3-antipyrine, m/z 192.04 (parent ion) to m/z 58.95 and 104.14 (both product ions)
